# High Starch in Diet Leads to Disruption of Hepatic Glycogen Metabolism and Liver Fibrosis in Largemouth Bass (*Micropterus salmoides*), Which is Mediated by the PI3K/Akt Signaling Pathway

**DOI:** 10.3389/fphys.2022.880513

**Published:** 2022-05-23

**Authors:** Liang Zhong, Hongli Liu, Haiqi Zhang, Weidong Zhang, Minghao Li, Ya Huang, Jiayun Yao, Xiaoli Huang, Yi Geng, Defang Chen, Ping Ouyang, Shiyong Yang, Wei Luo, Lizi Yin

**Affiliations:** ^1^ Department of Aquaculture, College of Animal Science and Technology, Sichuan Agricultural University, Chengdu, China; ^2^ Zhejiang Institute of Freshwater Fisheries, Hangzhou, China; ^3^ Department of Basic Veterinary, College of Veterinary Medicine, Sichuan Agricultural University, Chendu, China

**Keywords:** PI3K/Akt signaling pathway, high starch diets, hepatic fibrosis, liver damage, glucose metabolic disorders

## Abstract

Due to its special flavour and cheapness, starch is a source of nutrition for humans and most animals, some of whom even prefer to consume large amounts of starchy foods. However, the use of starch by carnivorous fish is limited and excessive starch intake can lead to liver damage, but the mechanism of damage is not clear. Therefore, in this study, two isonitrogenous and isolipid semi-pure diets, Z diet (0% starch) and G diet (22% starch), were formulated, respectively. The largemouth bass (*M*. *salmoides*) cultured in fiberglass tanks were randomly divided into two groups and fed the two diets for 45 days. Blood and liver were collected on day 30 and 45 for enzymology, histopathology, ultramicropathology, flow cytometry, and transcriptomics to investigate the damage of high starch on the liver of largemouth bass and its damage mechanism. The results showed that the high starch not affect the growth performance of largemouth bass. However, high starch caused a whitening of the liver and an increase in hepatopancreas index (HSI), aspartate aminotransferase (AST), and alanine aminotransferase (ALT) in the serum. Histopathological observations showed that high starch led to severe vacuolisation, congestion, and moderate to severe necrotizing hepatitis in the liver. The high starch intake led to a significant increase in postprandial blood glucose and insulin in serum of largemouth bass, promoting the synthesis and accumulation of large amounts of hepatic glycogen in the liver, leading to the loss of hepatocyte organelles and inducing liver fibrosis. Meanwhile, high starch induced the production of oxidative stress and promoted apoptosis and necrosis of hepatocytes. Transcriptome analysis revealed that there were 10,927 and 2,656 unique genes in the G and Z groups, respectively. KEGG enrichment analysis showed that 19 pathways were significantly enriched, including those related to glucose metabolism and cell survival. Network mapping based on enrichment pathways and differential expressing genes showed the emergence of a regulatory network dominated by PI3K/Akt signaling pathway. This indicated that the PI3K/Akt signalling pathway plays a very important role in this process, regulating the liver injury caused by high starch. Our results provide a reference for the mechanism of liver injury caused by high starch, and the PI3K/Akt signalling pathway could be a potential therapeutic target for liver injury caused by high starch.

## 1 Introduction

With the development of society, people’s material consumption level is constantly increasing and their demand for food is also put forward higher requirements, which leads to the increasing popularity of high-fat and high-carbohydrate food. And starch is one of the carbohydrates that, due to its special flavour and cheapness, is a source of nutrition for humans and most animals, some of whom even prefer to consume large amounts of starchy foods. However, studies have found that long-term consumption of high starch can place a serious burden on the body, leading to metabolic disorders and the emergence of various diseases, such as hyperlipidemia (Feng et al., 2015), obesity (Lee et al., 2011), and non-alcoholic fatty liver disease (NAFLD) (Vilà et al., 2014).

The liver is an important organ of the animal body and has very important physiological functions, such as nutrient metabolism, detoxification, and absorption (Hoekstra et al., 2013). Excessive intake of nutrients can therefore easily lead to damage to the liver. It was found that a high-fat diet led to severe steatosis, swelling of hepatocytes, and a significant increase in apoptosis in rats (Bede et al., 2020). A high-carbohydrate diet can lead to obesity, hepatitis, and liver oxidative stress in rats, which can lead to liver damage and even liver tumors (Panchal et al., 2011; Ip et al., 2014). Therefore, excessive carbohydrate intake is detrimental to the health of the liver.

The largemouth bass (*M*. *salmoides*), a typical carnivorous fish, is widely farmed in the world because of its high economic value. Numerous studies have shown that carnivorous fishes have a low utilization of starch (Zhang et al., 2018). Compared with herbivorous fish, carnivorous fish have lower amylase activity (Hidalgo et al., 1999). Moreover, the changes of glucose metabolism related enzymes such as glucose-6-phosphatase, pyruvate kinase and glycogen synthase in the liver of carnivorous fish were not obvious after consuming large amounts of starch, resulting in a longer period of hyperglycemia (Su et al., 2020). Therefore, a higher protein content and lower carbohydrate content are needed in the diet of carnivorous fish. However, in order to save feed cost, starch is widely used in aquatic feeds because of their cheap, easy-to-obtain and protein-saving function (Li et al., 2020). It was also found that high starch led to significant increases in serum glucose and insulin concentrations, aspartate aminotransferase (AST) and alanine aminotransferase (ALT) activities, significant increases in hepatic glycogen and malondialdehyde content, and significant hepatic vacuolization in Nile tilapia (*Oreochromis niloticus*) (Li et al., 2021). Likewise, high starch diets resulted in elevated plasma ALT, AST activity, glucose and insulin levels, and hepatic glycogen and muscle glycogen contents in largemouth bass. Expression of glucose metabolism genes, glucose-6-phosphate dehydrogenase, hexokinase, hexokinase, and insulin receptor substrate 1, was suppressed, and the liver was severely vacuolated and fibrotic (Lin et al., 2018; Yu et al., 2019; Zhang et al., 2019). Although there have been many studies on the effects of high starch on largemouth bass, most of these studies have focused on the effects of high starch diets on growth, liver tissue damage, and liver glycolipid metabolism in largemouth bass, and the underlying molecular mechanisms of liver damage caused by high starch are not clear.

Transcriptome is a powerful tool to gain insight into complex physiological responses and therefore it is widely used in various research fields (Shun et al., 2019). Transcriptome also has a wide range of applications in the field of aquaculture (Qian et al., 2014). For instance, Ouyang et al. (2021) found that Hepatopancreatic Necrosis Syndrome (HPNS) was associated with disruption of PDE-cAMP-related network through transcriptomic analysis. Some research already revealed some critical results in high carbohydrate issue by using transcriptome method. Such as, Blunt snout bream (*Megalobrama amblycephala*) fed high carbohydrate showed liver damage, and transcriptomic and qPCR analysis revealed upregulation of a large number of genes associated with the development of non-alcoholic fatty liver, such as *PI3K* and *Akt* (Prisingkorn et al., 2017).

The PI3K/Akt signaling pathway is an important pathway that is prevalent in all types of cells. It receives signals from insulin and is a major signaling pathway in insulin signal transduction (Zhan et al., 2012). Therefore, the PI3K/Akt signaling pathway mainly regulates glucose uptake, glycogen synthesis, and degradation. The normal functioning of PI3K/Akt pathway allows insulin to perform its normal hypoglycemic function. In addition, this signaling pathway also affects cell growth, cell cycle, and cell survival (Cantley, 2002). A variety of growth factors and other stimuli can activate PI3K/Akt signaling pathway (Zhao, 2013). The insulin is an important factor in activating this pathway. Insulin secreted by the organism binds to insulin receptors on the cell surface to phosphorylate insulin receptor substrate protein (IRS), which in turn activates the PI3K/Akt signaling pathway (Taniguchi et al., 2006). The phosphorylated Akt continues to activate downstream effector molecules, such as SGK3 and FOXO1, which in turn regulate physiological functions such as glucose metabolism, lipid metabolism, and cell survival in the organism (Boucher et al., 2014). It was found that activation of PI3K/Akt could improve hyperglycemia by reducing insulin resistance and increasing hepatic glycogen (Liu et al., 2019). In addition, several studies have confirmed that the PI3K/Akt signaling pathway is inextricably linked to liver fibrosis (Huang et al., 2011; Yang, 2017; Yu et al., 2019). However, whether the PI3K/Akt signaling pathway is involved in the regulation of high starch damage to the liver of largemouth bass has not been reported. Therefore, this study was conducted by feeding largemouth bass with high starch. The histopathology, enzymology, and transcriptomics were combined to investigate the mechanism of liver injury in largemouth bass. Our results provide a reference for the mechanism of liver injury caused by high starch.

## 2 Materials and Methods

### 2.1 Animal Ethics

All animal handling procedures were approved by the Animal Care and Use Committee of Sichuan Agricultural University, following the guidelines for animal experiments of Sichuan Agricultural University.

### 2.2 Diet Preparation

Formulation and proximate chemical composition of the trial diets are shown in Table 1 (Huang et al., 2021). Two (49% crude protein) and isolipidic (9% crude lipid) semi-purified diets were formulated with α- cassava starch (0 and 220 g/kg), named as Z diet and G diet, respectively. Briefly, all dry ingredients were crushed and sifted through 280 μm mesh, then weighed according to the ratio and mixed manually for 10 min. The mixed ingredients were transferred to a pelletizer (SYX62, Xiamen, China) and processed into 2 mm diameter pellets. All diets were air-dried at room temperature (23°C–30°C) and stored at −20°C until use.

**TABLE 1:** Formulation and proximate composition of the experimental diets.

Ingredients	Starch level in diets (g. kg^−1^)	
	Z	G
Fish meal^a^	490	490
Casein^a^	130	130
Soybean protein concentrate^a^	60	60
Soybean oil^a^	30	30
Soybean lecithin^a^	20	20
Yeast extract^a^	8	8
Ca(H_2_PO_4_)_2_ ^a^	10	10
Choline chloride^a^	3	3
Vitamin mixture^a^ ^,^ ^b^	8	8
Mineral mixture^a^ ^,^ ^c^	5	5
Carboxymethyl cellulose^a^	15	15
Lysine^a^	1	1
α- cassava starch^a^	0	220
Zeolite powder^a^	220	0
Proximate compositions (g. kg^−1^, dry matter)		
Crude protein	490	491
Crude lipid	82	91
Ash	301	103
Starch	13	224

a Supplied by Chengdu Sanwang Feed Ltd (Chengdu, China).

b Vitamin Premix (mg. kg^−1^ diet): vitamin A, 32.00; vitamin D3, 16.00; vitamin E, 351.83; vitamin K3, 30.03; vitamin C, 3288.80; vitamin B1, 19.77; vitamin B2, 60.00; vitamin B6, 36.43; vitamin B12, 24.00; niacinamide, 80.80; calcium-pan-tothenate, 75.10; folic acid, 6.73; inositol, 329.90; biotin, 32.00; L-carnitine, 102.03.

c Mineral mix (mg. kg^−1^ diet): FeSO_4_ (Fe), 70.33; MgSO_4_ (Mg), 351.33; CuSO_4_ (Cu), 8.00; ZnSO_4_ (Zn), 99.70; MnSO_4_ (Mn), 19.50; CoCl_2_ (Co), 19.37; Ca (IO_3_)_2_ (I), 50.17; Na_2_SeO_3_ (Se), 4.00.

The chemical composition analyses of the diets were conducted by standard methods (AOAC, 2005). Crude protein, crude lipid, and ash were determined by Kjeldahl method (N × 6.25) (Kjeltec 2300, FOSS, Hilleroed, Denmark), petroleum ether extraction (without acid hydrolysis) using Soxtec (Soxtec 2055; FOSS), and combustion at 550°C to constant weight in a muffle furnace (Shenyang Energy-saving Electric Furnace Factory, Shenyang, China). Optical rotation method was used to determine the starch content (spectropolarimeter WZZ-2B, Shanghai, China).

### 2.3 Feeding Management

Largemouth bass of similar weight (7.25 ± 1.67 g) and length (9.41 ± 0.51 cm) were purchased from a fish farm in Chengdu, Sichuan, China. The fish were acclimatized and fed Z diet in a big fiberglass tank for 2 weeks before experimentation. Then all fish were randomly distributed into 6 fiberglass tanks at 15 fish per tank after 24 h of starvation. Each of the two diets (Z and G diets) was randomly assigned to three tanks. The fish were fed twice a day (at 9 a.m. and 6 p.m.) for 45 days. The daily diet was fed at 8% of body weight. The daily amount of the diet was adjusted every 15 days by weighing the fish. During the experiment, the water was changed twice daily with advanced aeration after feeding. The largemouth bass were exposed to a light: dark cycle of 12 h:12 h. Water temperature was 24.9 ± 0.5°C, dissolved oxygen was >6.5 mg L^−1^, pH was 7–7.5, and ammonia-nitrogen was almost zero.

### 2.4 Sample Collection

The largemouth bass were sampled at 15, 30, and 45 days after the feeding experiment began. However, considering the short feeding period, the 15-day sample was only used for weighing and liver weight, measuring length, and calculating hepatopancreas index (HIS). All fish were fasted for 24 h prior to sampling, then the fish were anesthetized with MS-222 and were weighed and measured for body length. Then livers were stripped and weighed to calculate the HIS. Blood was collected from the tail vein, and blood from three fish was mixed into one sample. At 15 days, nine fish were collected for weighing and liver weight, measuring length, and calculating hepatopancreas index (HIS). At 30 and 45 days, five largemouth bass were used for histopathological and ultrastructural pathological observations, and six fish were used to determine glycogen, protein, triglycerides and were performed to Oil red O staining in the liver. In addition, nine fish livers from each group were taken for transcriptomic analysis and five fish livers were used for flow cytometry assays at 45 days.

### 2.5 Blood Physiology and Biochemistry

Blood was collected, centrifuged at low speed (3,000 rpm) for 10 min, and the supernatant was collected for blood physiological and biochemical assays. Serum glucose, glutathione aminotransferase (AST), alanine aminotransferase (ALT), triglyceride, malondialdehyde (MDA), superoxide dismutase (SOD), glutathione peroxidase (GSH-Px) and total protein as well as total protein, triglycerides and glycogen content in the liver were assayed using commercial kits (Nanjing Jiancheng Bioengineering Institute, Nanjing, China). Briefy, blood glucose was analyzed by glucose oxidase (GOD/POD) method, triglyceride by GPO/PAP (Khan et al., 1997), Glutathione aminotransferase (AST) and alanine aminotransferase (ALT) by the 2,4-dinitrophenylhydrazine method, malondialdehyde (MDA) by the thiobarbituric acid (TBA) method (Ma et al., 2019), superoxide dismutase (SOD) by the hydroxylamine method (Desrochers and Hoffert, 1983), total protein by the coomassie brilliant blue method, and liver glycogen by spectrophotometry (Li et al., 2018). Insulin was detected by ELISA assay kit (Shanghai Xin Yu Biotech Co., Ltd., Shanghai, China). All enzyme assays were performed in triplicate.

### 2.6 Histological Analysis

The livers of five fish sampled after 30 and 45 days were fixed in 10% neutral buffered formalin for 48 h. Then the liver tissues were also trimmed into cassettes, rinsed overnight, dehydrated in graded ethanol solutions, cleared in xylene, and embedded in paraffin wax. Sections of 4 μm for hematoxylin and eosin (H&E) staining, periodic acid Schiff (PAS) staining, Sirius Red staining, and masson staining were prepared prior to microscopic analysis. Three mosson stained photographs with the same magnification were randomly selected from each group and analyzed for relative quantification using ImageJ.

The frozen sections of liver tissue were fixed in 4% paraformaldehyde for 15 min after reheated and dried. Oil red O was added on the slides and stained for 8–10 min, and rinsed with tap water, differentiated with 75% ethanol and re-stained with hematoxylin. Sealed sections were observed by optical microscope (CX 33, Olympus, Japan).

The degree of vacuolar degeneration, necrosis and congestion was scored with reference to the modified grade scoring system established by Baums et al. (2013). Histological changes were assessed using a score ranging from 1 to 7, depending on the extent of the lesion: (1) unchanged; (3) mild; (5) moderate; and (7) severe.

### 2.7 Electron Microscopy

Fresh liver tissues were sampled at a thickness of 2 mm^3^ and were fixed in fixative (2.5% glutaraldehyde in pH 7.4 cacodylate buffer) for 2 h at room temperature. The liver tissues were washed three times in PBS and post-fixed in 1% osmium tetroxide. The samples were dehydrated through ascending concentrations of alcohol and post-embedded in Araldite. Cross-oriented ultra-thin sections were cut and stained with uranyl acetate and lead citrate. Images were acquired on a HITACHI HT7700 transmission electron microscope (Tokyo, Japan).

### 2.8 Flow Cytometry

Liver tissues of largemouth bass were collected in ice-cold PBS. The livers were immediately minced to form a cell suspension and filtered through a 300-mesh nylon screen. Cells were washed twice with cold PBS (4°C), and the cell pellet was resuspended at a concentration of 1 × 10^6^ cells/mL in PBS. The cell cycle was detected using 1,000 μl cell suspension after co-incubation with 500 μl of PI/Rnase staining Buffer (Cycle kit, United States, Item No: BD) for 30 min. Reactive oxygen species (ROS) was detected by DCFH-DA staining (ROS Assay Kit, China Item No: 88-5930-74) and cell apoptosis was determined by using Annexin V-FITC stain (Apoptosis Kit, Australia, Item No: BMS500fi-300). After sample pre-treatment, detection was performed by Cytoflex flow cytometer (Backman, United States). Modfit and Kaluza 2.1 software were used to analyze the data.

### 2.9 Transcriptomics (RNA-Seq) Analysis

#### 2.9.1 RNA Library Construction and Sequence

Three largemouth bass in each group were used for RNA-seq. Total RNA was extracted with reference to the kit instructions (Foregene Company, Limited, China), and its concentration and purity were detected by Nanodrop 2000 (Thermo, Waltham, MA, United States) and the integrity was detected by Agilent Bioanalyzer 2,100 (Agilent, Palo Alto, United States). The mRNA was enriched with magnetic beads with Oligo (dT) and fragmented with a fragmentation buffer. cDNA was synthesized using the fragmented mRNA as a template. In order to select cDNA fragments of preferentially 370–420 bp in length, the library fragments were purified with AMPure XP system (Beckman Coulter, Beverly, United States). Then PCR amplification was performed and the PCR products were purified again using AMPure XP beads to obtain the final library. After the library was constructed, the library was initially quantified using a Qubit 2.0 Fluorometer and diluted to 1.5 ng/μL, followed by the detection of the insert size of the library using an Agilent 2,100 bioanalyzer. The qRT-PCR was performed to accurately quantify the effective concentration of the library (effective library concentration above 2 nM). The transcriptome sequencing was conducted using Illumina HiSeq™ 2,500. Sequencing data has been uploaded to the NCBI database (accession number: SRR15959224 and SRR15960096).

#### 2.9.2 Transcriptome Quality Control, Assembly, and Functional Annotation

Raw data (raw reads) of fastq format were firstly processed through in-house perl scripts. Clean data (clean reads) were obtained by removing adapter reads, ploy-N and low-quality reads from raw data. At the same time, Q20, Q30, and GC content of the clean data were calculated. The obtained high-quality reads were used for the downstream analyses. Transcriptome assembly was performed by using Trinity software (v2.4.0) (Grabherr et al., 2011). Then the list of read counts was obtained by clustering and quantifying the transcripts (Corset v4.6). Transcript splicing quality was assessed using BUSCO software. BLAST software was used to compare unigenes with Nr, Nt, Pfam, KOG/COG, Swiss-Prot, KEGG, and GO databases (e value ≤ 1e −5), to obtain the annotation information.

#### 2.9.3 Differential Expression Genes Analysis

Differential expression genes (DEGs) analysis was performed using the DESeq2 R package (1.20.0). Genes with |log 2 (Fold Change) | > 1 and an adjusted *p*-value <0.05 were identified as differentially expressed. The differential genes were then annotated into GO or KEGG databases. GOseq (1.10.0) and KOBAS software (v2.0.12) were used to perform GO functional enrichment analysis and KEGG pathway enrichment analysis on the differential genes (corrected *p*-value<0.05).

### 2.10 Validation of Quantitative Real Time RT-PCR

To validate the transcriptome data, the quantitative real time PCR (qRT-PCR) was used to detect sample RNA from the transcriptome. Six differential expression genes were selected to assay. The primers were designed using Primer Premier 6 (Table 2). Complementary DNA (cDNA) was synthesized from 1 μg of RNA using a PrimeScript RT reagent kit with gDNA Eraser (TaKaRa). qPCR was performed using a SYBR green real-time PCR kit (TaKaRa) and a Thermo Cycler (BioRad, Hercules, CA, United States).

**TABLE 2:** Transcriptome verification gene primer sequences.

Target gene	Abbreviation	Primer (5′-3′)	Amplicon size (bp)	Tm (°C)
Insulin receptor substrate-2	*IRS2*	F: CCG​TCC​GCG​CAG​TAA​AAG​CC	198	65.0
		R: ATC​ATG​GTG​CCG​TCG​CCC​TC		
Epidermal growth factor receptor	*EGFR*	F: GCCCTGTCTATCAATGCC	261	55.4
		R: GCT​ACC​ACG​TTT​AGT​TCG​TC		
Lactate dehydrogenase	*LDH*	F: AGTGTGACAGCCAACTCC	202	60.0
		R: TCAGCTTCCAGGCCACGT		
Glucose transporter-1	*GLUT-1*	F: AAT​CGC​TTT​GGA​AGG​AGG​A	175	59.5
		R: CAC​CCA​CAT​ACA​TTG​GCA​CA		
Forkhead box transcription factor O1	*FOXO1*	F: TGC​TGC​TCT​TTT​CCT​GGA​GA	228	61.4
		R: TTC​TCA​GAG​GTT​CCG​TGC​AT		
Carnitine palmitoyltransferase1A	*CPT1α*	F: AGC​CCC​ACC​CCA​ACC​TAC​CAG	280	65.0
		R: CGG​CCC​TCA​CGG​AAT​AAA​CGC		
β-actin	*β-actin*	F:AAAGGGAAATCGTGCGTGAC	136	61.0
		R:AAGGAAGGCTGGAAGAGGG		

When performing qPCR reactions, a 10 μl reaction mixture was used, including 5 μl SYBR Green PCR Master Mix, 3 μl diethylpyrocarbonate-treated water, 0.4 μl of forward primer, 0.4 μl of reverse primer, 0.2 Rox II, and 1 μl cDNA. The reaction procedure was as follows: 3 min at 95°C for 1 cycle, then samples were amplified for 40 cycles at 95°C for 10 s, a melting temperature based the specific primer pair for 30 s, followed by 10 s at 95°C and 72°C for 10 s. To distinguish between specific and non-specific reaction products, a melting curve was obtained at the end of each run. Relative changes in mRNA transcript expression were calculated from qPCR results using the 2^−ΔΔCT^ method (Livak and Schmittgen, 2002), using β-actin as an internal reference gene.

### 2.11 Statistical Analysis

The results were expressed as the mean value and standard deviation (S.D.). One-way ANOVA was used to compare the differences between different times within the same group in growth performance, and independent sample *t*-test was used to analyze the significance of other differences. Statistical analyses were performed using IBM SPSS 20.0 software (IBM Corp., Armonk, NY, United States), and a *p*-value < 0.05 was considered significant.

## 3 Results

### 3.1 Effects of High Starch Diets on Growth Performance and Liver of Largemouth Bass

The effects of high starch diets on the growth performance and liver of largemouth bass were showed in Table 3. After 45 days of culture, the high starch diet did not lead to death of largemouth bass. And there was no significant difference in final body weight (FBW), final body length (FBL), weight gain rate (WGR), and specific growth rate (SGR) in group G compared with group Z (*p* > 0.05), but FBW, FBL, and WGR increased with the increase of breeding time, while SGR showed the opposite trend (*p* < 0.05). However, the liver weight of largemouth bass fed G diet increased significantly with the increase of feeding time, while the HSI showed the opposite trend (*p* < 0.05), which might be due to the increase of body weight led to the decrease of HSI. However, the liver weight and HSI of largemouth bass fed on G diet were significantly higher than those fed on Z diet (*p* < 0.01). This indicates that the high starch diet significantly increased the HSI of largemouth bass and thus caused damage to the liver of the fish.

**TABLE 3:** Effects of high starch diet on the survival and growth of largemouth bass.

		Z group	G group
SR (%)		100	100
IBW (g)		7.12 ± 1.61	7.35 ± 1.11
IBL (cm)		9.34 ± 0.51	9.31 ± 0.45
FBW (g)	15 d	11.06 ± 1.52^a^	12.91 ± 2.62^a,^**
	30 d	17.99 ± 3.95^b^	19.89 ± 2.70^b^
	45 d	27.20 ± 4.93^c^	28.19 ± 5.56^c^
FBL (cm)	15 d	9.93 ± 0.39^a^	10.16 ± 0.53^a^
	30 d	11.30 ± 0.56^b^	11.48 ± 0.46^b^
	45 d	13.02 ± 0.71^c^	13.02 ± 0.66^c^
WGR (%)	15 d	0.63 ± 0.30^a^	0.86 ± 0.54^a^
	30 d	1.80 ± 1.07^b^	1.95 ± 0.66^b^
	45 d	2.95 ± 1.10^c^	3.01 ± 0.96^c^
SGR (%/d)	15 d	0.094 ± 0.123^b^	0.116 ± 0.201^b^
	30 d	0.081 ± 0.115^a,b^	0.087 ± 0.002^a^
	45 d	0.066 ± 0.004^a^	0.068 ± 0.004^a^
LW (g)	15 d	0.20 ± 0.24	0.39 ± 0.13^a,^**
	30 d	0.14 ± 0.41	0.43 ± 0.16^a,b,^***
	45 d	0.24 ± 0.06	0.65 ± 0.19^b,^**
HIS (%)	15 d	1.79 ± 1.76^b^	3.10 ± 1.07^b,^**
	30 d	0.79 ± 0.15^a^	2.17 ± 0.89^a,^***
	45 d	0.88 ± 0.07^a^	2.31 ± 0.50^a,^***

SR (survival rate, %) = 100 × final fish number/initial fish number; WGR (weight gain rate, %) = 100 × (FBW−IBW)/IBW, where FBW and IBM are the final and the initial body weight of fish; Specific growth rate (SGR, %) = [ln (mean final weight)−ln (mean initial weight)/45 days] ×100; HIS (%) = LW/IBM, where LW is the liver weight of fish; IBL and FBL: the initial and final body length; **p < 0.01 or ***p < 0.001 represents highly significant differences between fed Z and G diets at the same time. Lowercase letters represent differences between different times fed the same diet. The “a, b, c” represents differences between different times fed the same diet.

### 3.2 High Starch Diets Disrupt Liver Function in Largemouth Bass

**FIGURE 1:**
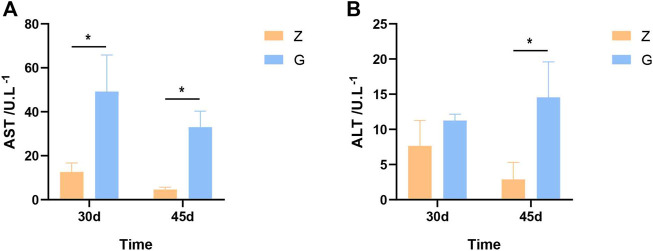
Liver damage markers in largemouth bass. **(A,B)** AST and ALT after 30 and 45 days, respectively (*p < 0.05).

To investigate whether the high starch diet caused liver damage in largemouth bass, markers of liver damage were examined at 30 and 45 days. The aspartate aminotransferase (AST) and alanine aminotransferase (ALT) of largemouth bass fed the G diet were significantly higher than those of largemouth bass fed the Z diet at both 30 and 45 days (*p* < 0.05) (Figure 1). This indicates that high starch feeds can cause liver damage and disrupt liver function in largemouth bass.

### 3.3 High Starch Diets Disrupt Cell Morphological Integrity in Liver of Largemouth Bass

**FIGURE 2:**
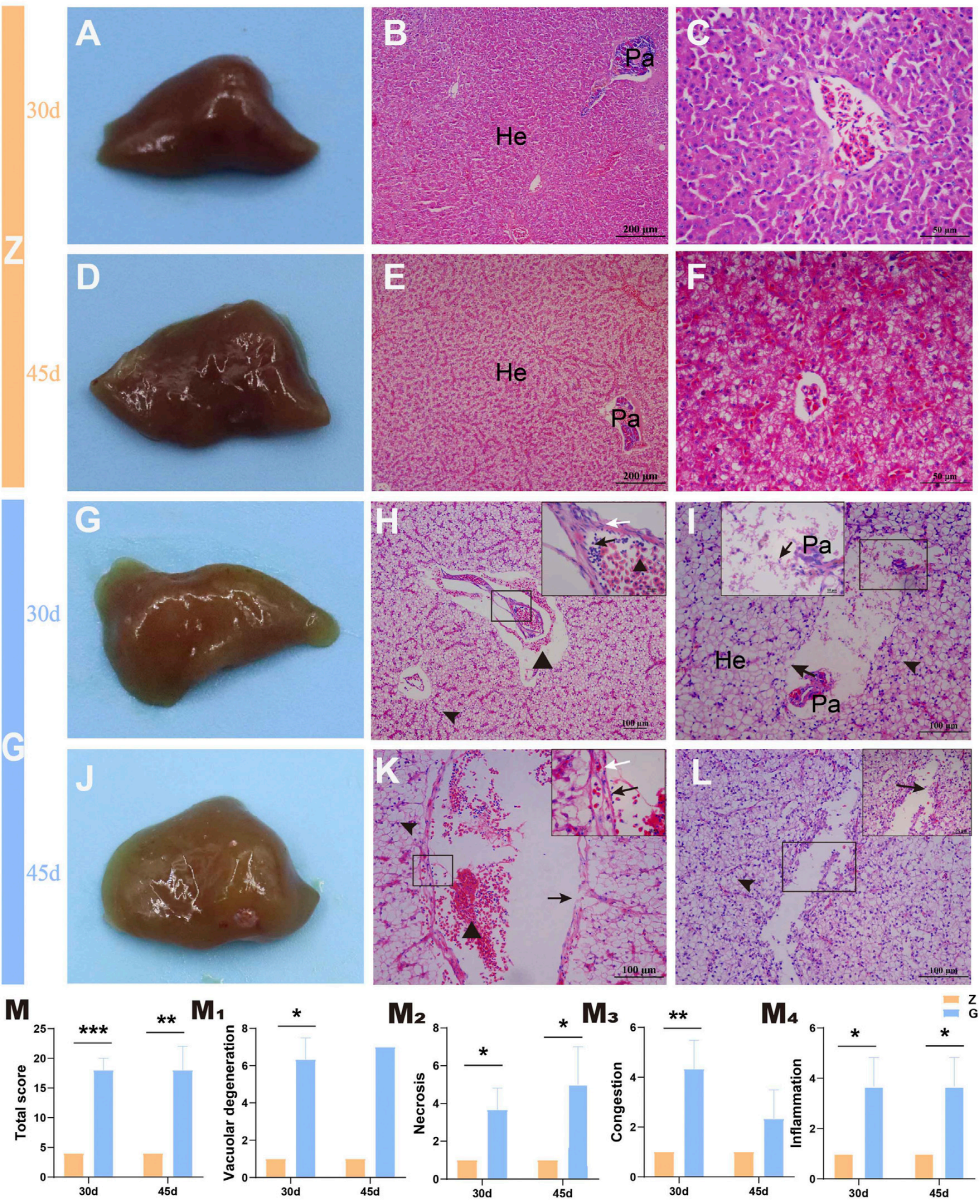
Gross pathology and histopathological observations of the largemouth bass liver (H&E staining). **(A–C)** and **(D–F)** The liver of largemouth bass fed Z diet at 30 and 45 days, respectively. **(A**,**D)** The livers of largemouth bass fed Z diet were ruddy. **(B,C)** and **(E,F)** Hepatocyte morphology with no obvious morphological changes. **(G–I)** and **(J–L)** The liver of largemouth bass fed G diet at 30 and 45 days, respectively. **(G**,**J)** The livers of largemouth bass fed Z diet were visibly white. **(H**,**K)** Severe vacuolation of hepatocytes (arrowhead), congestion of the hepatic sinusoids (triangle), thickening of the vascular endothelium (white arrow), and accompanying inflammatory cell infiltration (black arrow). **(I**,**L)** Vascular endothelial cell and hepatocyte necrosis, pancreatic atrophy, and pancreatic cell necrosis and detachment (black arrow). **(M)** Total liver histopathological score. **(M**_**1**_ **–M**_**4**_ **)** The histopathological scores for vacuolar degeneration, necrosis, congestion, and inflammation, respectively. He, hepatocytes; Pa, pancreas (*p < 0.05, **p < 0.01, and ***p < 0.001).

To further observe the tissue damage caused by high starch diets to the liver of largemouth bass, histopathological observation was performed. Livers of largemouth bass fed the Z diet were ruddy both at 30 and 45 days (Figures 2A,D). Histopathological observations revealed intact hepatocyte morphology with no obvious morphological changes. The majority of hepatocyte was lined with a double-layered. The sinusoidal capillaries between the hepatic plates were narrowed in an irregular sinusoidal pattern. Straight capillaries connected through perilobular vessels to the centrolobular vessels. The hepatocytes were polyhedral or rounded with an eosinophilic cytoplasm and a rounded nucleus located in the centre of the cell. Pancreatic tissue was diffusely distributed in the hepatic parenchyma, separated from it by a thin layer of connective tissue (Figures 2B,C,E,F). However, the livers of largemouth bass fed the G diet were whitish (Figures 2G,J), and histopathological observations showed severe vacuolar degeneration, enlarged hepatocytes with blank cytoplasm and borderline shifted nuclei, necrosis and thickening of hepatic vascular endothelial cells, and congestion in hepatic sinusoids with inflammatory cell infiltration; as well as pancreatic atrophy, with pancreatic cells exhibiting marked necrosis and detachment (Figures 2H,I,K,L). According to the liver histopathology score, feeding a high starch diet resulted in severe damage to the liver of largemouth bass, which manifested as severe vacuolar degeneration, congestion and moderate to severe necrotizing hepatitis (*p* < 0.05) (Figures 2M–M_4_).

### 3.4 High Starch Diet Damages Largemouth Bass Liver by Promoting Glycogen Deposition

**FIGURE 3:**
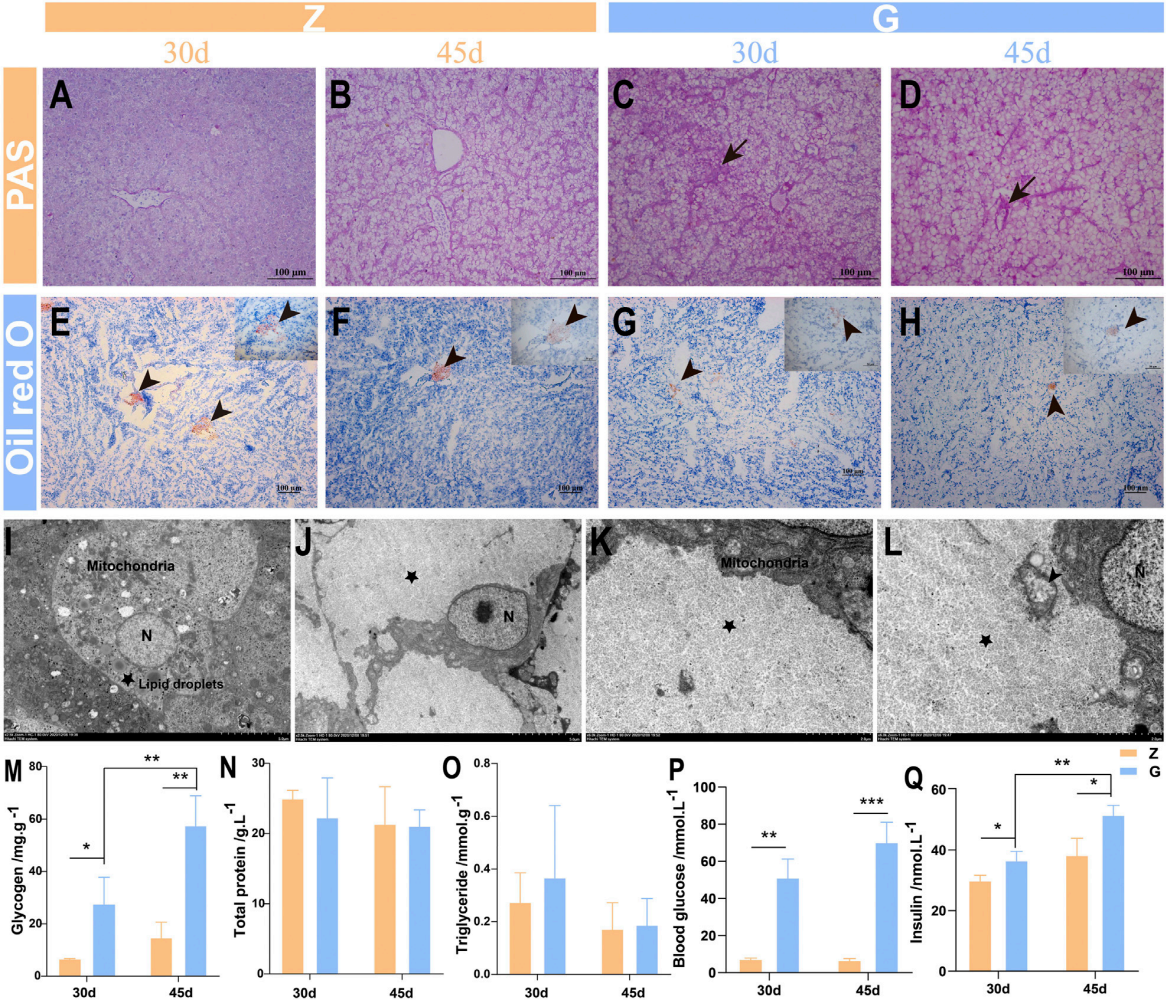
Liver glycogen and fat detection of largemouth bass. **(A–D)** PAS staining. **(A,B)** The liver of largemouth bass fed the Z diet for 30 and 45 days, respectively. **(C,D)** The liver of largemouth bass fed the G diet for 30 and 45 days, respectively Glycogen accumulation (arrow). **(E–H)** Oil red O staining. **(E,F)** The liver of largemouth bass fed the Z diet for 30 and 45 days, respectively. **(G,H)** The liver of largemouth bass fed the G diet for 30 and 45 days, respectively Fat deposition (arrowhead). **(I–L)** Ultrastructural pathology of the largemouth bass hepatocytes after 45 days of feeding the two diets. **(I)** Largemouth bass fed the Z diet had an intact hepatocyte morphology with abundant organelles and small amounts of glycogen and lipid droplets deposited. **(J–L)** Large amounts of glycogen deposition (pentagram), reduced organelles, swollen mitochondria with the break and disappearance of cristae (arrowhead) in the hepatocytes of largemouth bass fed a G diet. **(M–O)** Glycogen, triglycerides and total protein were determined in the livers of largemouth bass, respectively. **(P,Q)** Determination of blood glucose and insulin levels in the serum of largemouth bass (*p < 0.05, **p < 0.01, and ***p < 0.001).

To further investigate the causes of liver vacuolation, PAS and Oil red O staining were performed. The PAS staining results showed that only a small amount of glycogen was stored in the livers of largemouth bass fed the Z diet (Figures 3A,B), while more glycogen particles appeared in the livers of those fed the G diet (Figures 3C,D). Oil Red O staining revealed only a small amount of fat in the livers of fish fed with Z and G diets (Figures 3E–H). Subsequently, the structure of the ultramicroscopic material in the hepatocytes was further observed by electron microscopy. Hepatocytes of largemouth bass fed the Z diet were morphologically intact, with abundant organelles such as mitochondria, endoplasmic reticulum, and lysosomes, and a small accumulation of lipid droplets and glycogen (Figure 3I). However, the hepatocytes of the largemouth bass fed the G diet had a large amount of glycogen particles deposited in the hepatocytes (Figures 3J–L), resulting in the nucleus displaced to one side (Figure 3J). The number of organelles in the cells was reduced and only few mitochondria was seen (Figure 3K), and the mitochondrial morphology was damaged with the break and disappearance of cristae (Figure 3L). Tests on liver glycogen also showed that feeding the G diet significantly promoted glycogen storage in the liver (*p* < 0.05) (Figure 3M). The triglyceride and total protein assays showed no difference between the two diets (*p* > 0.05) (Figures 3N,O). The feeding of high starch diet to largemouth bass led to a significant increase in blood glucose (*p* < 0.01) (Figure 3P), which in turn induced a significant increase in insulin secretion (*p* < 0.05) (Figure 3Q), which may be responsible for the massive glycogen accumulation. This suggests that feeding a high starch diet is damaging the liver by increasing the accumulation of glycogen in the livers of largemouth bass.

### 3.5 High Starch Diet Causes Liver Fibrosis in Largemouth Bass

**FIGURE 4:**
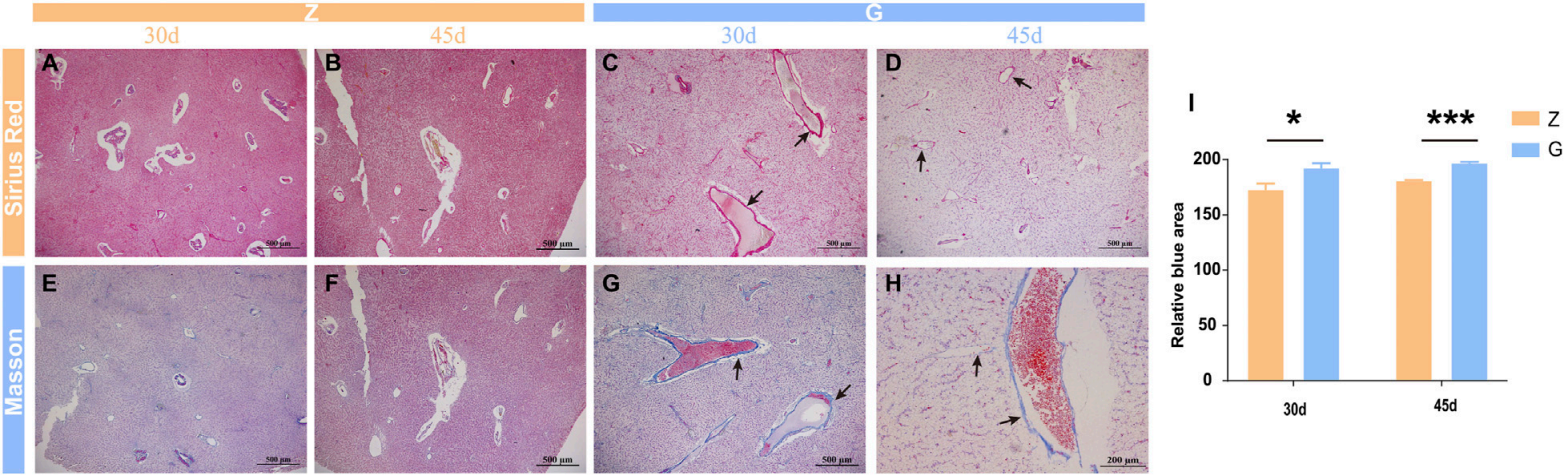
Sirius red and Mosson staining in largemouth bass liver. **(A–D)** Sirius red staining fibroblasts stain red (arrow). **(E–H)** Mosson staining; fibroblasts stain light blue (arrow). **(I)** Relative quantitative analysis of Mosson staining (*p < 0.05, ***p < 0.001).

Studies in mammals have found that the accumulation of large amounts of glycogen in the liver leads to severe fibrosis in the liver (Yi et al., 2012; Liu et al., 2014; Pursell et al., 2018), but it is unclear whether the same results occur in fish, however structural and morphological features of thickened vessel walls are visible on liver sections staining with H&E, so liver fibrosis in largemouth bass was observed by special staining. Sirius red staining revealed no significant fibrosis in the livers of largemouth bass fed the Z diet (Figures 4A,B), whereas the livers of those fed the G diet showed significant periportal fibrosis (Figures 4C,D). Similar results were obtained with Mosson staining (Figures 4E–H). Relative quantification of Masson staining showed significant fibrosis in the livers of largemouth bass fed a high starch diet (*p* < 0.05) (Figure 4I). This suggested that glycogen accumulation in the liver due to feeding a high starch diet leads to liver fibrosis.

### 3.6 High Starch Diet Damages Largemouth Bass Liver by Promoting Apoptosis and Necrosis of Hepatocytes

**FIGURE 5:**
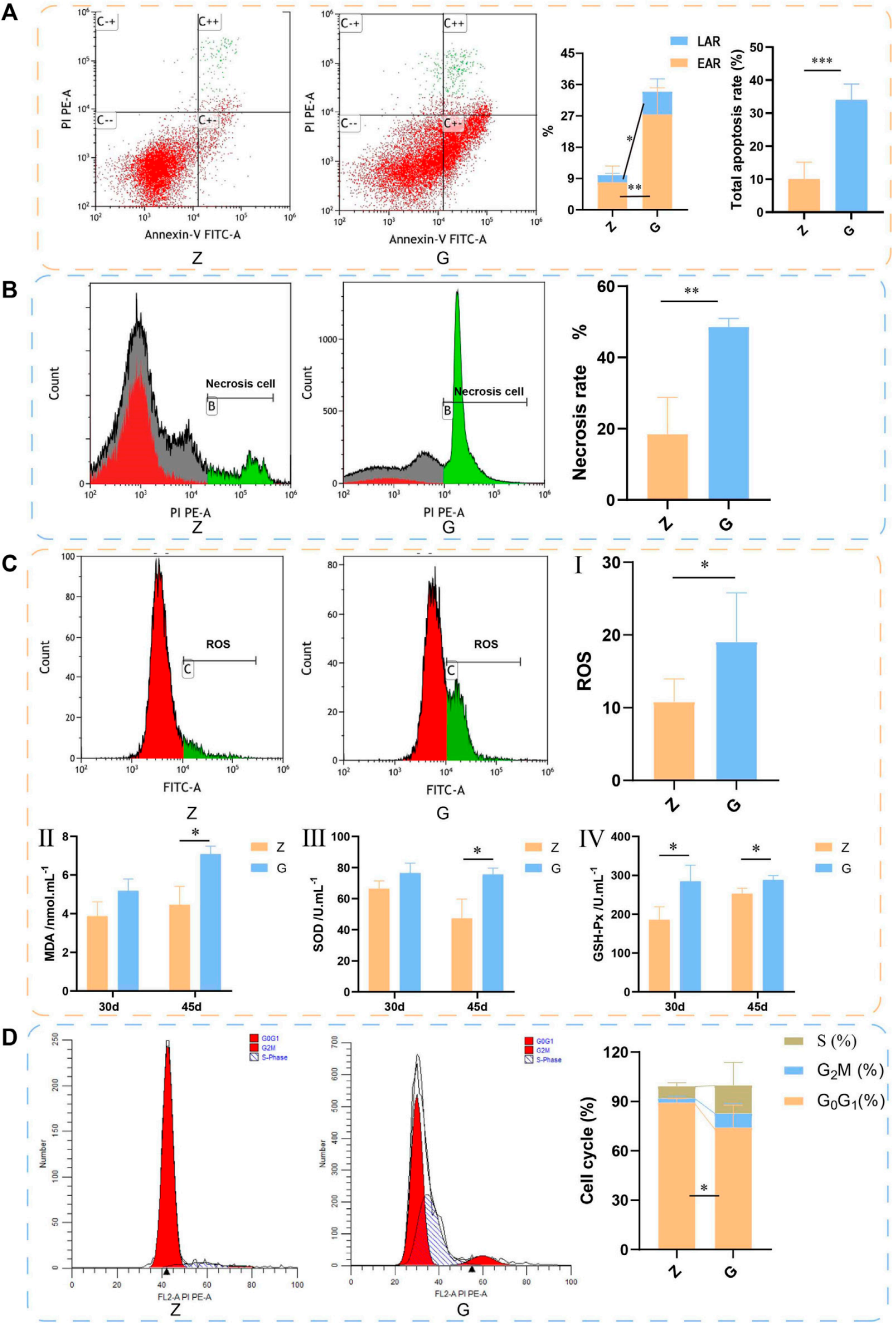
The livers of largemouth bass fed with 45 days by flow cytometry assay. **(A)** Apoptosis detected by flow cytometry. Annexin-V binds to phosphatidylserine on the surface of cells to detect early apoptosis. PI is a DNA dye that enters the nucleus and binds to DNA to indicate late apoptosis. C−−: live cells; C+−: early apoptotic cells; C++ late apoptotic cells; C−+: mechanically damaged cells. LAR: early apoptosis rate, EAR: late apoptosis rate. **(B)** Necrosis detected by flow cytometry. **(C)** ROS detected by flow cytometry. (II–IV) Detection of serum MDA, SOD, and GSH-Px. **(D)** Cell cycle detected by flow cytometry (*p < 0.05, **p < 0.01, and ***p < 0.001).

Since significant hepatocyte necrosis was found in H&E staining of liver sections, the effect of a high starch diet on the survival of largemouth bass hepatocytes was further examined by flow cytometry. The results are shown in Figure 5. The early apoptosis rate (EAR), late apoptosis rate (LAR), and total apoptosis rate of largemouth bass hepatocytes fed on a G diet (27.42% ± 7.62%, 6.59% ± 3.66%, and 34.01% ± 4.80%, respectively) were significantly higher than those fed on a Z diet (7.89% ± 4.70%, 2.08% ± 0.52%, and 9.98% ± 5.16%, respectively) (*p* < 0.05) (Figure 5A). Meanwhile, the necrosis rate of livers was significantly higher in largemouth bass fed the G diet (G: 48.53% ± 2.41%; Z: 18.44% ± 10.27%) (*p* < 0.01) (Figure 5B). Cell survival would be closely related to oxidative stress of the organism. The detection of ROS in the liver was also found to be significantly higher in largemouth bass fed the G (19.04% ± 6.76%) diet than in those fed the Z diet (10.82% ± 3.14%) (*p* < 0.05) (Figure 5CI). And the lipid peroxidation product MDA content was significantly higher (*p* < 0.05) (Figure 5CII). Interestingly, the activities of SOD and GSH-Px were also significantly higher (*p* < 0.05) (Figures 5CIII,IV). This seemed to indicated that the organism was expressing more antioxidant enzymes to eliminate the adverse effects of oxidative stress. In addition, the cell cycle was also examined by flow cytometry. The number of cells in the G0G1 phase of the livers of largemouth bass fed the G diet was significantly lower (G: 74.19% ± 13.61%; Z: 89.34% ± 2.49%) (*p* < 0.05), while the number of cells in the S and G2M phases did not change significantly (Figure 5D). These results suggest that feeding a high starch diet induces oxidative damage to the body, which in turn promotes apoptosis and necrosis of hepatocytes and damages the liver.

### 3.7 High Starch Diet Alters the Gene Expression Pattern of Largemouth Bass Liver

**FIGURE 6:**
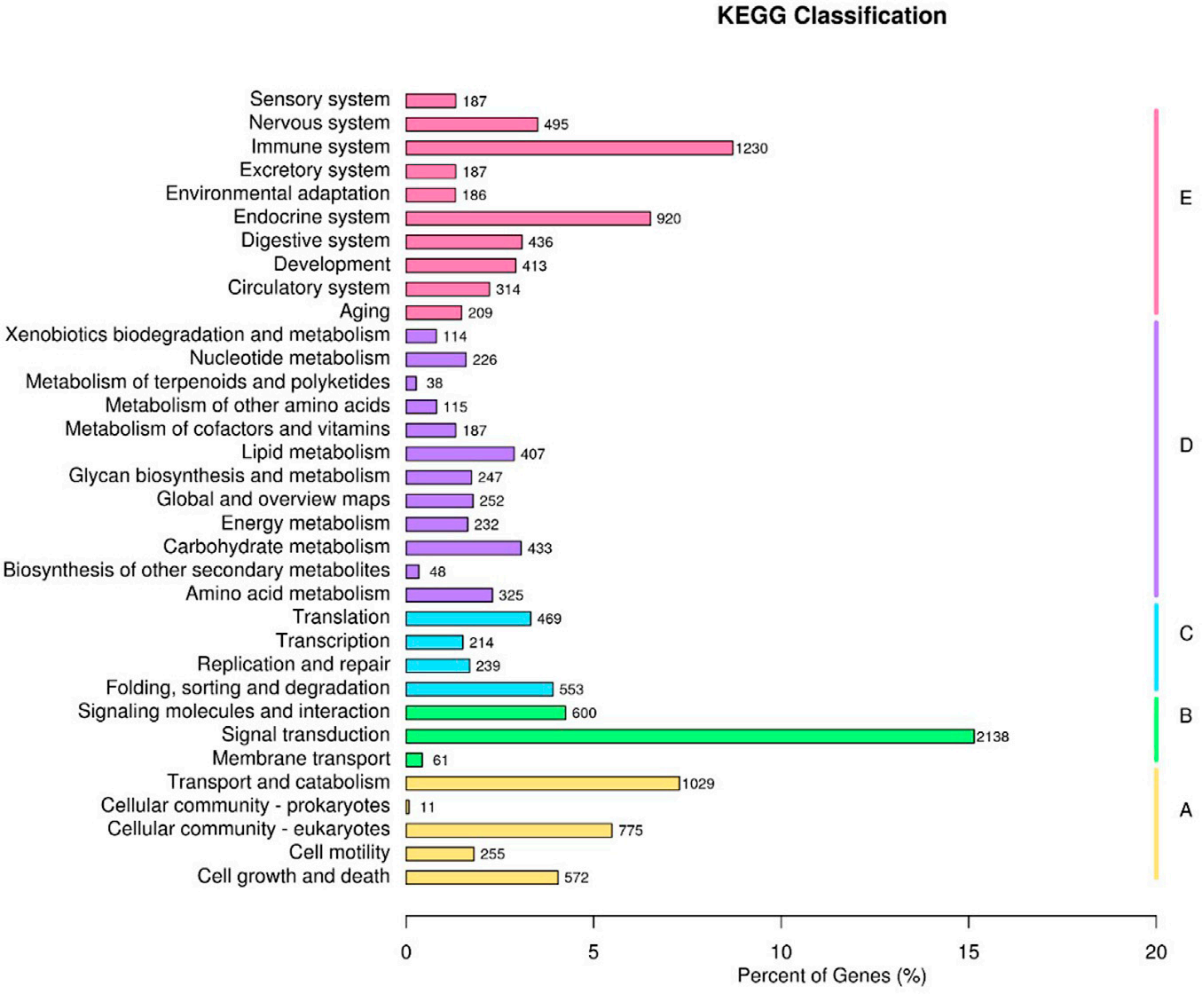
KEGG functional classifications. **(A)** Cellular Processes, **(B)** Environmental Information Processing, **(C)** Genetic Information Processing, **(D)** Metabolism, **(E)** Organismal Systems.

**FIGURE 7:**
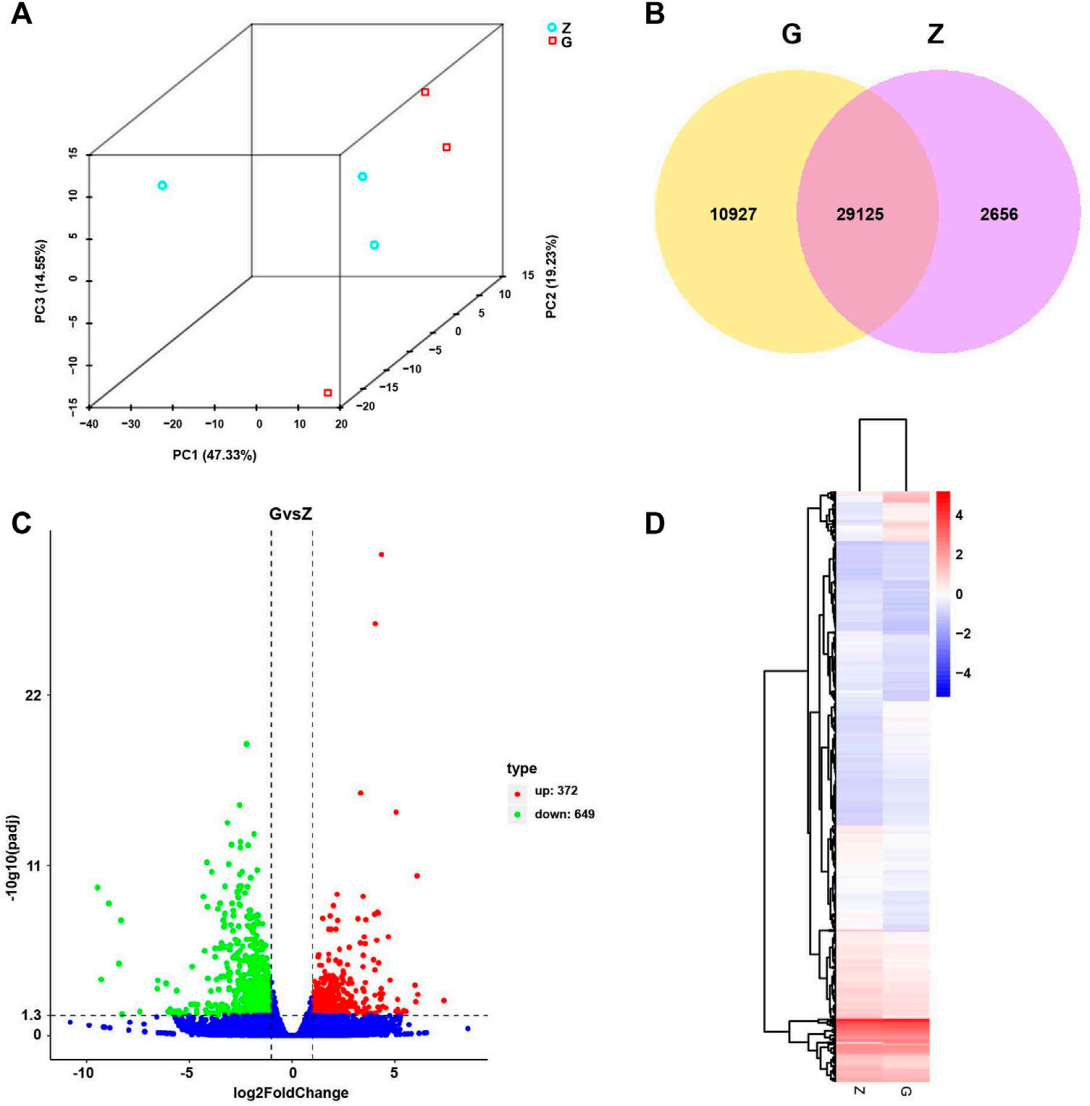
Principal Component Analysis (PCA), Differential Expression (DE) analysis, and Hierarchical Clustering (HC) of fed with high starch diet (G) compared with fed with free-starch diet (Z). **(A)** PCA analysis of all samples showed a clear separation between group Z and group G along PC1. **(B)** Venn diagram of unique and shared genes. **(C)** Volcano plot of differential genes in group G compared with group Z. Red dots represent upregulated genes and green dots represent downregulated genes. **(D)** Hierarchical clustering (HC) analysis of differential genes in group Z and group G. High expression is shown in red and low expression in blue.

To elucidate the pathogenesis of starch-induced liver injury, gene expression profiles were performed on livers of largemouth bass fed two diets for 45 days by RNA-seq analysis. After quality control of the transcriptome assay data, high quality data were obtained with clean bases greater than 6G, an error tolerance of 0.03%, and a Q2 greater than 94% (Table 4). The splicing assembly by Trinity software produced 43,350 unigenes, and the average length of unigenes was 1,526 bp (N50 = 2,946) (Supplementary Figure S1). To obtain comprehensive gene function information, gene function annotation was performed for seven databases, with the highest annotation rate in the Nt database and the lowest in KOG (Supplementary Figure S2). The annotation results showed that the KEGG functional classifications involved in the genes were classified into Cellular Processes, Environmental Information Processing, Genetic Information Processing, Metabolism, and Organismal Systems, among which the Metabolic had the most genes involved in carbohydrate metabolism (Figure 6). Principal component analysis (PCA) showed that the two groups were clearly separated, with a variability of 47.33% (PC1) (Figure 7A). 29,125 genes were expressed in the G and Z groups, and 10,927 and 2,656 genes were unique, respectively, which indicated that the high starch diet caused a significant increase in gene expression in the liver of largemouth bass (Figure 7B). A total of 1,038 genes were significantly differentially expressed in group G compared to group Z, of which 373 genes were upregulated and 665 genes were downregulated (Figure 7C). The clustering analysis of differential genes further showed the effect of high starch diet on the liver gene expression pattern of largemouth bass (Figure 7D). To verify the accuracy of the transcriptome data, qRT-PCR was used to detect differential expression genes. In general, the results of RNA-seq were mainly consistent with those of qRT-PCR, indicating that the transcriptome data are reliable (Supplementary Figure S3).

**TABLE 4:** Quality control information for transcriptome samples.

Sample	Raw reads	Clean reads	Clean bases (G)	Error rate (%)	Q20 (%)	Q30 (%)	GC (%)
Z1	21890597	21392855	6.42	0.03	96.11	89.83	48.83
Z2	21462418	20946424	6.28	0.03	96.17	89.96	48.76
Z3	22082740	21651534	6.50	0.03	94.83	87.54	47.51
G1	22157921	21565469	6.47	0.03	95.58	88.9	49.33
G2	23443286	23084195	6.93	0.03	94.37	86.74	48.18
G3	22662206	22179522	6.65	0.03	94.81	87.6	48.38

### 3.8 PI3K/Akt may be Potential Mechanisms of High Starch Damage to the Liver

**FIGURE 8:**
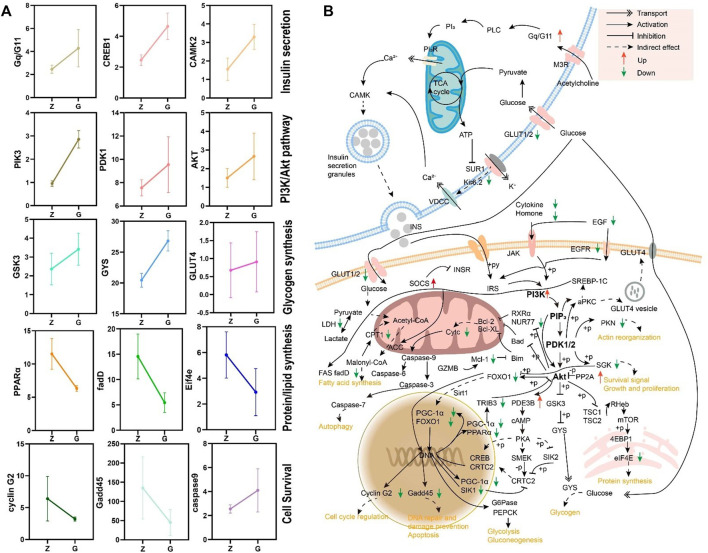
**(A)** Expression patterns of genes in the KEGG enriched pathway. **(B)** A regulatory network based on transcriptomic data for high starch injury in the liver of largemouth bass.

The differential expression genes (DEGs) were further enriched into the KEGG database for pathway enrichment analysis. The results revealed that 19 signaling pathways were significantly enriched (*p* < 0.05), including those related to glucose metabolism and cell survival (Table 5). Further analysis revealed significantly different expression patterns of genes related to glucose metabolism and cell survival in the livers of largemouth bass fed G feed compared to Z feed (Figure 8A). Then the network of the effect of high starch on the liver was mapped according to the KEGG enrichment pathway and DEGs (Figure 8B). The results showed that a PI3K/Akt-dominated regulatory network emerged in the livers of largemouth bass fed the G feed compared with those fed the Z feed. The feeding of high starch led to the production of hyperglycaemia in largemouth bass, which in turn induced an increase in insulin secretion by pancreatic β-cells. This was confirmed in previous results. PI3K received signals from high levels of insulin and was led to increased expression. The upregulation of PI3K expression might facilitate glucose uptake by the liver and promote glycogen synthesis, while inhibiting protein and fatty acid synthesis. At the same time, high PI3K expression may also activate the apoptotic pathway in hepatocytes and affect cell survival. Therefore, the abnormal expression of PI3K may lead to disruption of hepatic glucose metabolism, cell survival and cell proliferation pathways in largemouth bass, ultimately manifesting as pathological phenomenas such as hepatic glycogen accumulation, hepatocyte necrosis and hepatic fibrosis. And the previous findings confirmed the reliability of the transcriptomic data. Therefore, we speculate that the PA3K/Akt signalling pathway may be a potential molecular mechanism regulating liver injury in largemouth bass under high starch feeding.

**TABLE 5:** KEGG analysis of DEGs obtained by transcriptome sequencing.

KEGG pathway	Description	Number of differential genes	*p*-value
ko04640	Hematopoietic cell lineage	11	0.000341177
ko04972	Pancreatic secretion	15	0.000442171
ko00270	Cysteine and methionine metabolism	9	0.001072538
ko04974	Protein digestion and absorption	13	0.001521002
ko00564	Glycerophospholipid metabolism	12	0.009676582
ko04975	Fat digestion and absorption	7	0.014742483
ko00670	One carbon pool by folate	4	0.016060399
ko03320	PPAR signaling pathway	10	0.018371058
ko04010	MAPK signaling pathway	19	0.022586385
ko00760	Nicotinate and nicotinamide metabolism	5	0.025301473
ko00910	Nitrogen metabolism	3	0.033902126
ko04977	Vitamin digestion and absorption	5	0.038096205
ko00100	Steroid biosynthesis	4	0.039271805
ko04630	Jak-STAT signaling pathway	10	0.040293061
ko00430	Taurine and hypotaurine metabolism	2	0.043708749
ko04922	Glucagon signaling pathway	11	0.044627801
ko05202	Transcriptional misregulation in cancer	13	0.049408923
ko05230	Central carbon metabolism in cancer	8	0.049669884
ko05211	Renal cell carcinoma	8	0.049669884
ko04213	Longevity regulating pathway—multiple species	7	0.0616776

## 4 Discussion

As one of the common carbohydrates, starch is an inexpensive source of energy, and therefore has become a common ingredient in modern intensive farming feeds. The optimal dietary starch levels could help to facilitate feed production and promote growth and feed efficiency in farmed fish (Mingchun et al., 2011; Zhou et al., 2015). However, excessive dietary starch can cause many adverse effects on carnivorous fish with poor starch utilization. Tilapia fed with excessive starch showed a significant decrease in weight gain rate and specific growth rate, and a significant increase in blood glucose and liver glycogen content (Han et al., 2021). High starch diets elevated blood glucose and liver glycogen content and decreased liver triglyceride content in tropical hybrid catfish, but did not affect their growth performance (Okamura et al., 2019). Largemouth bass fed a high starch diet showed a significant decrease in growth performance, a significant increase in liver/muscle glycogen content, plasma glutamic aminotransferase (ALT), glutamic aminotransferase (AST) activity, glucose, and insulin content, and an opposite trend in triglyceride content, along with cell enlargement, nuclear disappearance, and severe vacuolization in the liver (Lin et al., 2018; Zhang et al., 2020). In addition, high starch diets led to abnormal lipid metabolism in carnivorous fish, with large amounts of lipids deposited in the liver, such as barramundi (*Lates calcarifer*) (Wade et al., 2020) and European seabass (*Dicentrarchus labrax L.*) (Viegas et al., 2016). This suggests that different fish behave differently in response to high starch diets, with excess glucose stored in the liver as glycogen or as triglycerides (Li et al., 2022). In this study, although high starch diet did not affect the growth performance of largemouth bass, it significantly promoted liver whitening and increased hepatopancreas index. Histopathological observations showed significant vacuolization, inflammation, necrosis, and congestion. Meanwhile, serum glucose, insulin, AST, and ALT activities were significantly enhanced and hepatic glycogen accumulated heavily, while no significant changes were seen in triglycerides. This suggests that the high starch did cause liver damage in largemouth bass. This was similar to the findings of Amoah et al. (2008).

Glycogen accumulation disease is a disorder of glucose metabolism caused by defects in key enzymes in the glycogen catabolic process. Defects in key enzymes lead to ineffective glycogen breakdown in the liver, resulting in the accumulation of large amounts of glycogen in the liver, which in turn leads to liver enlargement and even to liver fibrosis and cirrhosis, eventually leading to liver failure (Markowitz et al., 1993; Liu et al., 2014). Although there are no reports of defects in key enzymes in glycogenolysis in fish, large accumulation of hepatic glycogen has been widely confirmed under high carbohydrate feeding (Liu et al., 2021; Su et al., 2021). And excessive hepatic glycogen accumulation can impair hepatocyte function, which in turn can negatively affect the growth and health status of fish (Liu et al., 2021). Similar results were obtained in the present study, where high starch diets led to a significant increase in hepatic glycogen content, which may be the pathological basis for the appearance of vacuolation. Meanwhile the excessive accumulation of glycogen squeezed the nucleus and other organelles, causing the disappearance of organelles within the hepatocytes, which inevitably affected the survival of hepatocytes. Therefore, a significant increase in the number of apoptotic and necrotic hepatocytes was detected by flow cytometry. In addition, studies have reported that high starch feeding resulted in significant oxidative stress in the liver (Lin et al., 2018), and similar to this the levels of ROS and MDA were significantly increased in this study, which may be a factor leading to apoptosis and necrosis of hepatocytes (Conde de la Rosa et al., 2015). However, it is interesting to note that the antioxidant enzymes (SOD and GSH-Px) activities were not reduced, which seems to indicate that the antioxidant enzyme system of the organism has not been completely damaged and the organism is still trying its best to reduce the damage caused by oxidative stress. But anyway, the high starch diet can cause oxidative stress damage to largemouth bass and affect their health.

The PI3K/AKT signalling pathway has an important role in physiological and pathological processes such as cell metabolism, cycling and apoptosis. It is commonly believed that this signalling pathway plays a vital role in insulin signalling and insulin-mediated glucose metabolism pathways (Taniguchi et al., 2006). In the presence of insulin, the insulin receptor (IR) phosphorylates insulin receptor substrate proteins (IRS proteins), which further activate the PI3K/AKT pathway. Activated Akt exerts protein kinase activity to phosphorylate a variety of downstream molecules, which in turn regulate a variety of glucose metabolism-related enzymes and glucose transporters, exerting a regulatory role in glucose metabolism (Li et al., 2017). It has been found that the PI3K/AKT pathway can be regulated to improve hyperglycaemia symptoms in type 2 diabetic patients (Kuai et al., 2016). PI3K/AKT pathway is also widely involved in the regulation of cell cycle, apoptosis and necrosis (Mikami et al., 2010; Xie et al., 2018). Therefore, the PI3K/AKT pathway is considered to be an attractive target for cancer therapy (Bellacosa et al., 2005). In this study, 24 h postprandial blood glucose was significantly increased in largemouth bass fed a high-starch diet, which induced an increase in insulin secretion and activated the PI3K/AKT signalling pathway. We hypothesize that the activation of this signalling pathway could promote the expression of its downstream glucose metabolism genes (e.g., GSK-3 and GYS), which led to the synthesis and accumulation of large amounts of hepatic glycogen in the livers of largemouth bass, resulting in significant vacuolization and fibrosis of the liver; on the other hand, it led to increased apoptosis and necrosis of hepatocytes by inhibiting the expression of anti-apoptotic genes (e.g., MCL1) and cell cycle regulation and DNA repair genes (e.g., Gadd45 and cyclin G2), thus leading to a marked increase in apoptosis and necrosis. Damage to hepatocytes stimulates the activation of hepatic stellate cells (Benyon and Iredale, 2000; Zhao, 2013), which may contribute to the development of liver fibrosis. And the regulation of cell cycle genes resulted in a decrease in the number of G0/G1 phase cells and a trend towards an increase in the number of S and G2/M phase cells, which may stem from the body’s own regulation by promoting cell proliferation to compensate for apoptotic and necrotic cells.

## 5 Conclusion

This study confirms that high starch feeds lead to increased postprandial blood glucose, liver bleaching, massive accumulation of hepatic glycogen and oxidative stress, promoting apoptosis and necrosis of hepatocytes, which in turn damages the liver and affects the health of largemouth bass. Combined with transcriptomic analysis, the PI3K/Akt signalling pathway was shown to play an important role in liver injury caused by high starch. Our results provide a reference for the mechanism of liver injury caused by high starch, and the PI3K/Akt signalling pathway may become a potential therapeutic target for liver injury caused by high starch.

## Data Availability

The datasets presented in this study can be found in online repositories. The names of the repository/repositories and accession number(s) can be found below: https://www.ncbi.nlm.nih.gov/, SRR15959224 and SRR15960096.
