# Supplementary material for: High Starch in Diet Leads to Disruption of Hepatic Glycogen Metabolism and Liver Fibrosis in Largemouth Bass (Micropterus salmoides), Which is Mediated by the PI3K/Akt Signaling Pathway
Source: Front Physiol. 2022 May 23;13:880513. doi: 10.3389/fphys.2022.880513 (PMC9168315; doi:10.3389/fphys.2022.880513)
Supplement: Supplementary file 1 [file Table1.DOCX]

**Supplementary data**


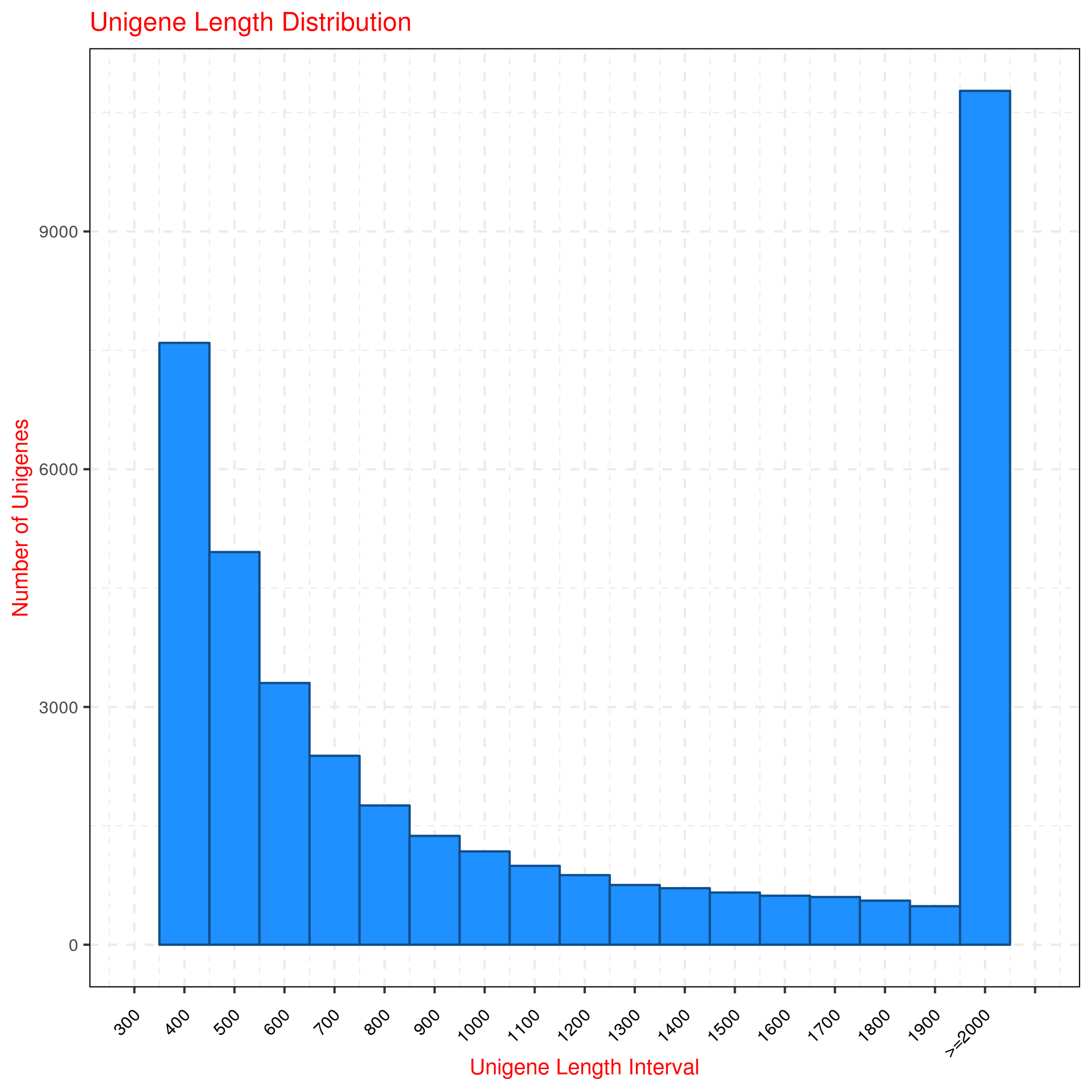


Figure S1. Number and length distribution of unigenes


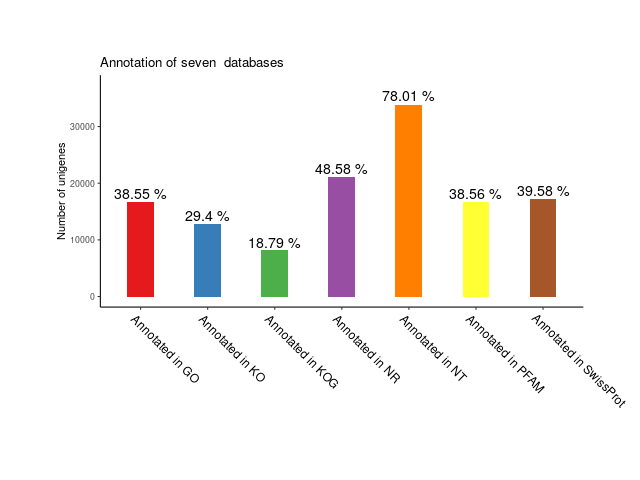


Figure S2. Gene annotation in the seven databases


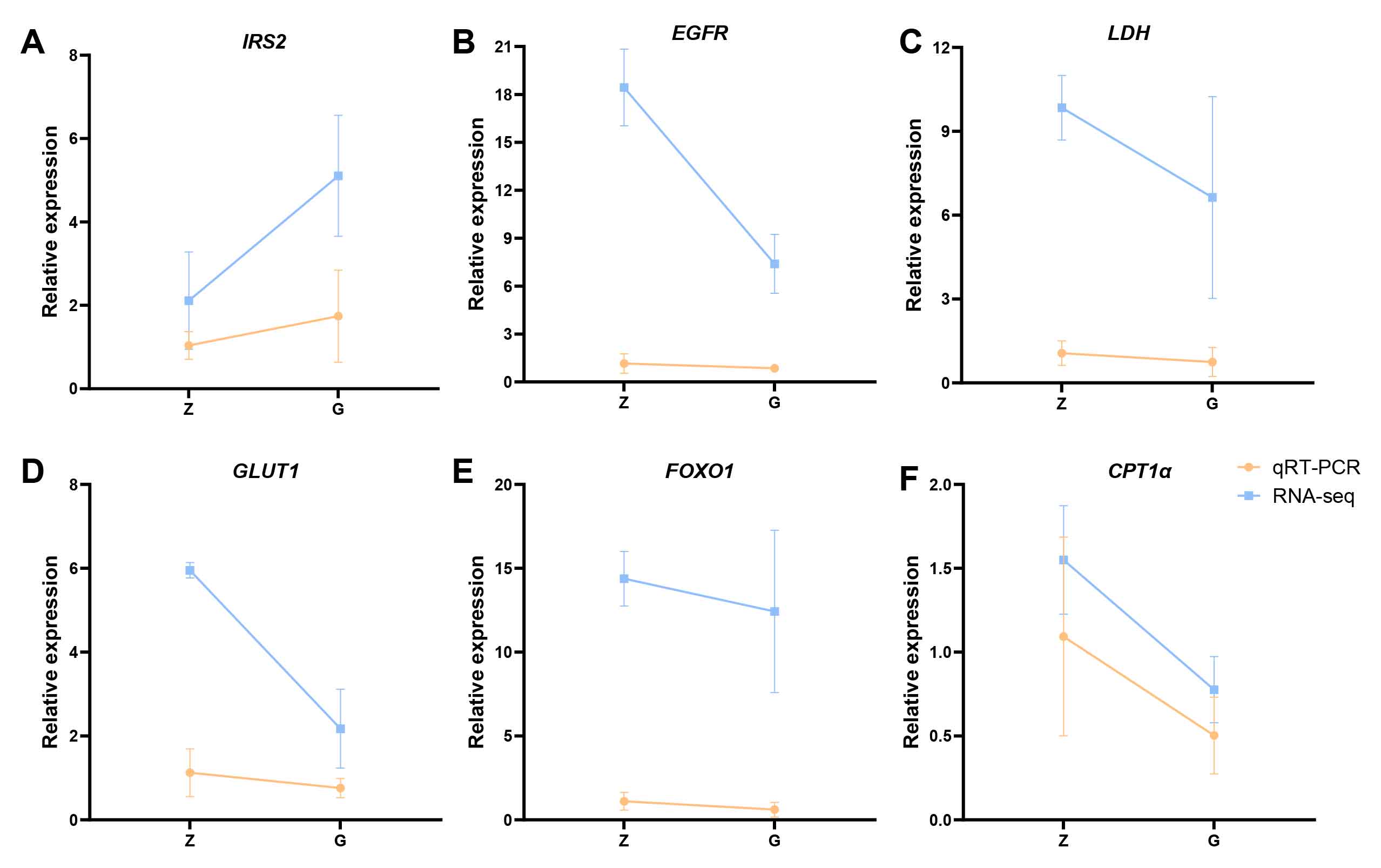


Figure S3. QRT-PCR was used to detect differential expression genes in the transcriptome data. The results of RNA-seq were mainly consistent with those of qRT-PCR.
